# The conversing military chaplain: time allocation, task salience, and competencies among Swedish military chaplains

**DOI:** 10.3389/fpsyt.2026.1816636

**Published:** 2026-04-15

**Authors:** Jan Grimell

**Affiliations:** Department of Sociology, Faculty of Social Sciences, Umeå University, Umeå, Sweden

**Keywords:** competencies, military Chaplains, military soul care, moral injury, pastoral care, spiritual care

## Abstract

Military chaplaincy is an established yet multifaceted practice within military organizations and is exposed to particular stressors such as the use of violence, ethical dilemmas, loss, and existential vulnerability. This study examines how a Swedish normative framework for Military Soul Care (ACCES: advisory role, command and crisis support, ceremonies, education, and soul care conversations) interacts with Swedish military chaplains’ own experiences of what they perceive as most important and meaningful in their mission. The empirical material consists of qualitative questionnaire data collected in 2025 from 50 military chaplains. The material was analyzed using an abductive approach and organized thematically. The results show that conversations constitute the task to which the greatest amount of time is devoted across both main categories of military chaplains, and that conversations are understood broadly, ranging from informal everyday interactions to confidential individual soul care conversations. Various forms of ceremonies and crisis support related to death and grief were experienced as particularly meaningful and reflect a clearly articulated priestly identity. Educational tasks varied between categories, with time constraints and organizational priorities limiting opportunities depending on context. A central finding is that presence within the organization, aimed at building relationships and trust, emerges as a decisive prerequisite and contributes to many chaplains working beyond their contracted hours. The importance of presence is not explicitly articulated in the ACCES framework but rather permeates the mission implicitly. Against the backdrop of a changed security environment, the findings illustrate that ecclesial priestly competencies related to crisis response, death, grief, and funeral expertise constitute a particularly vital resource in situations of crisis and war.

## Introduction

Military chaplaincy is an established yet inherently multifaceted form of pastoral and spiritual care within armed forces and international military operations (1, 2). Military chaplains (from here on MCs) operate in contexts characterized by distinctive and often cumulative stressors, including exposure to violence, ethical and moral dilemmas, loss, separation, and profound existential vulnerability (3–6). Their mandate encompasses, though is not limited to, the provision of pastoral conversations, crisis intervention, education, ceremonial and ritual practices, and existential and spiritual guidance for military personnel. Such care is offered both in everyday service and in connection with deployments, training exercises, and particularly demanding or traumatic events (1). Military chaplaincy is not directed solely toward individuals in acute distress; it also serves a preventive and sustaining function that supports the long-term psychological, social, and existential well-being of service members, as well as veterans experiencing moral injury and related forms of spiritual distress (5, 7–11). This practice is marked by sustained presence within the everyday life of the military and by care that is often delivered in close proximity to operational settings and units (1, 12–15).

MCs bring to this work a broad and integrative competence profile (1). They typically have professional training in theology, religious or humanistic studies, and pastoral ministry, frequently complemented by education in pastoral and spiritual care and counseling, crisis support, and ethics (7, 16, 17). In addition to these foundational competencies, effective chaplaincy in military settings requires contextual knowledge of organizational structures, leadership dynamics, military culture, and the psychosocial and existential conditions that shape military life. In practice, chaplains move fluidly among multiple roles: offering confidential pastoral conversations, advising commanders and leaders, participating in crisis-response systems, and contributing to ethical reflection, moral formation, and values-based dialogue within units (1, 5, 18–20). Their work is therefore not necessarily confined to confessional or explicitly religious activities. Rather, it is characterized by an interprofessional and integrative orientation in which spiritual, psychological, moral, and organizational dimensions of care intersect.

At the same time, military chaplaincy is not shaped solely by individual competencies and vocational commitments, nor by personal freedom in relation to the assignment as such; rather, it is embedded within explicit institutional, legal, and normative frameworks (1, 21, 22). These frameworks consist of formal mandates, policy documents, and regulatory structures that define the scope, responsibilities, and limitations of chaplaincy within the military organization (1, 16, 23, 24). They delineate, among other issues, chaplains’ relationships to the chain of command, confidentiality and privileged communication, confessional identity, and collaboration with other care providers such as medical, psychological, and mental-health professionals (16, 23, 25). While such frameworks provide legitimacy, protection, and institutional support for pastoral care, they may also generate tensions between professional autonomy, pastoral confidentiality, and organizational or operational demands (16, 20, 22). Moreover, formal frameworks may reflect practitioners’ lived experiences of what is essential for meaningful and effective pastoral care to varying degrees. Understanding military chaplaincy as a pastoral practice therefore requires analytical attention to both chaplains’ embodied, relational competencies and the structural conditions within which their care is enacted.

### Purpose

The purpose of this article is to examine how the Swedish military framework of formally defined chaplaincy tasks interacts with chaplains’ lived experiences of what they perceive as most important and meaningful in fulfilling their pastoral vocation. The study draws on qualitative data collected in 2025 and includes 50 Swedish MCs, representing approximately half of the rapidly expanding chaplaincy population in Sweden.

### Framing Swedish military chaplaincy: contextual conditions and the ACCES framework

Until the year 2000, Sweden maintained a formal state church, when a parliamentary decision established a separation between the state and the Church. Although the Church of Sweden no longer holds state-church status in a legal sense, it continues to be governed through political church elections in which Sweden’s traditional political parties remain influential (26). These elections take place every four years and follow the same principles of representation at national, regional, and local levels as general elections to Parliament, regions, and municipalities (27). The Church of Sweden occupies a distinctive position within Swedish society and, on behalf of the state, serves as the authority responsible for funeral services in most parts of the country (28). Beyond this practical and administrative role, the Church contributes significantly to the maintenance of cultural traditions, ritual practices, and value orientations that continue to shape Swedish social life (29–32). Currently, slightly more than half of the Swedish population—approximately 5.4 million individuals—are members of the Church of Sweden (33).

As a Lutheran church, the Church of Sweden is historically intertwined with the sixteenth-century Reformation and the formation of the Swedish nation-state (27). Over time, the Church has become a well-established institutional and cultural presence, best understood as deeply embedded within Swedish society rather than positioned as a distinct or marginal religious actor (31). In this context, priests from the Church of Sweden have, in an unbroken historical continuity spanning nearly five centuries, been present within the Swedish Armed Forces, offering pastoral and existential support to military personnel (34).

In contemporary practice, MCs in the Swedish Armed Forces are almost exclusively ordained priests of the Church of Sweden. This arrangement reflects historical continuity, cultural expectations, and the regulatory and normative frameworks governing military service. The appointment of MCs is shaped by multiple criteria, including advanced academic theological education, compatibility with the Armed Forces’ core values (for example, commitments to LGBTQ rights), extensive pastoral experience encompassing parish and funeral services, rigorous security vetting, and adherence to established institutional norms and professional standards (27).

With few exceptions, MCs are employed by the Church of Sweden and serve within the Armed Forces either as part of their ordinary clerical positions (for example, at regiments, air wings, and bases) and, in the case of the Home Guard, alongside their civilian employment. Depending on the nature and scope of a particular assignment, a corresponding employment agreement may be established with the Armed Forces; nevertheless, the Church of Sweden typically remains the primary employer. During extended international deployments, priests are granted leave from their ecclesial positions and are temporarily employed by the Swedish Armed Forces for the duration of the mission.

The professional practice carried out by MCs within the Armed Forces is referred to as Military Soul Care (militär själavård). This form of priestly practice is organized through a structured framework comprising five core functions, summarized by the acronym ACCES (29):

1. Advisory role—providing ethical, moral, and existential counsel to commanders and advising the Church of Sweden on issues related to defense readiness and war preparedness;
2. Commander and crisis support—offering sustained support to leaders and personnel during periods of heightened stress, crisis, and operational strain;
3. Ceremonies—conducting field services, prayers, memorials, and other significant ritual practices;
4. Education—providing education in ethics and moral reasoning, as well as instruction concerning war graves and related responsibilities; and.
5. Soul care conversations—engaging in confidential, existentially oriented pastoral conversations grounded in professional secrecy.

Within the doctrinal structure of the Armed Forces, Military Soul Care is situated in the pillar of moral factors, thereby contributing to overall defense readiness and operational capacity. Moral factors refer to the human and ethical dimensions of military practice, including resolve, courage, endurance, cohesion, leadership, education, training, preparation, and ethical conduct (35).

Importantly, Military Soul Care is not primarily framed as a matter of individual religious entitlement or as an expression of the Armed Forces’ legal obligation to provide religious services. Instead, it operates according to a functional and relational logic oriented toward strengthening resilience under conditions of crisis and war, while simultaneously supporting the will to defend and, when necessary, to engage in combat (36).

In conclusion, Military Soul Care in Sweden is shaped by the ACCES framework and embedded within a distinct national and cultural context. Located within the doctrinal domain of moral factors, it contributes to the Armed Forces’ overall capacity for military action by supporting moral formation, existential clarity, and spiritual well-being. The overarching aim is to strengthen the inner resilience and ethical orientation of military personnel, particularly under wartime conditions. Drawing on theological and ecclesial competence, advanced pastoral skills, and an in-depth understanding of military culture, training, and operational realities, military chaplains provide an existentially informed form of Military Soul Care—rather than a narrowly religious or devotional practice—through the ACCES model (29, 37; for a full explanation of ACCES, see 27).

## Method

The empirical material used in this article is drawn from a study of Swedish MCs conducted in March 2025 within the framework of a research project commissioned by the Chief of Chaplains of the Swedish Armed Forces. The overall purpose of the study was to contribute to a deeper and more up-to-date understanding of Swedish military chaplaincy, a professional field that remains underexplored in qualitative research.

To this end, a qualitative questionnaire was employed. This approach both aligned with earlier Swedish studies and allowed for the collection of data that combined breadth with analytical depth (38, 39). The format made it possible to include a relatively large number of participants while still generating rich, reflective, and open-ended responses comparable to those produced in qualitative interviews. Anonymity was a central feature of the design and was intended to facilitate candid accounts of personal experiences, reflections, and emotional dimensions of chaplaincy work.

The study underwent ethical review and received formal approval from the Swedish Ethical Review Authority (Reference number: 2024-08417-01).

### Focus of analysis

The qualitative questionnaire was structured into several thematic sections, each combining targeted questions with open-ended items intended to capture participants’ experiences and reflections on various dimensions of their professional practice. In the present article, the analysis is limited to the thematic section addressing professional duties, as presented below. One additional question was incorporated from another section of the questionnaire, as it was deemed to correspond particularly well with an overarching perspective on professional duties:

- In relation to the duties of a MC—Advisory support, Commander & Crisis support, Ceremonial duties, Education, and Soul care conversations (ACCES)—how would you describe the way your time is distributed among these responsibilities? (Please estimate in percentages or describe in another suitable way.)
- Which of these duties do you consider most important within your role, and why?
- To what extent does this (see previous question) correspond with your experiences of meaning and stimulation in your work?
- If there are any duties that you perform rarely or not at all, what do you believe are the reasons for this?
- Do you work as a MC beyond the percentage allocated to this role in your official position, and if so, why?
- What are the most important ecclesiastical and knowledge-based resources or tools at your disposal in your capacity as a MC?

Responses to this question constitute the empirical basis for the findings presented in this article.

### Data collection procedure

Data collection took place during the Swedish military chaplains’ annual training days, held on 12–13 March 2025 at the Swedish Armed Forces Headquarters in Stockholm. As electronic devices such as computers and mobile phones were not permitted within the facility, the questionnaire was administered in paper form.

The study was introduced orally to the participants, after which they received written information about the project together with an informed consent form. Participation was entirely voluntary. Respondents were allotted 1 hour and 40 minutes to complete the questionnaire—approximately equivalent to the duration of an in-depth research interview.

To safeguard anonymity, no identifying information was collected in the questionnaires themselves. Consent forms were gathered separately, ensuring that individual responses could not be linked to specific participants.

### Categories of MCs in Sweden

MCs in Sweden serve in several distinct organizational roles. With only a small number of exceptions, they are ordained Lutheran priests within the Church of Sweden.

The first category comprises priests employed by the Church of Sweden who, alongside their ordinary parish duties, serve as MCs at regiments, air bases, naval stations, and garrisons within their local pastoral areas. The proportion of time devoted to military chaplaincy varies, typically ranging from 20 to 75 percent, with the remaining time allocated to parish responsibilities. In this article, this group is referred to as *Regiments, Air Bases, Naval Stations, and Garrisons MCs*.

The second category consists of MCs affiliated with Home Guard battalions. These battalions are composed of part-time service members who retain civilian occupations but can be mobilized at short notice. MCs in this category are full-time parish priests who perform an additional 8–10 mandatory duty days per year with their assigned battalion. These duties are usually carried out under employment with the Home Guard Battalion—that is, the Swedish Armed Forces—rather than within their parish employment. This group is referred to here as *Home Guard Battalion MCs*.

A third category operates at the regional command level. These regional MCs are attached to one of Sweden’s five military regions—North, Central, West, South, and Gotland—and a significant portion of their work is conducted at this level. Regional staffs play a central role in leading operations during national crises and in supporting civilian society. In this category, the Swedish Armed Forces acts as the formal employer.

In addition to these three main categories, there are certain exceptions and supplementary arrangements, which have been discussed elsewhere (see 40).

### Participants

The participants’ background and characteristics are presented in **Table 1** below.

**Table 1:** Sample characteristics.

Category	Description
Total number of participants	50
Gender distribution	32 men, 18 women
Age distribution	Majority aged 35–55; fewer under 35 and over 55
Service roles	17: regiments, air bases, naval stations, and garrisons 24: Home Guard battalions 3: sensitive positions (not specified) 6: role not disclosed (anonymity concerns)
Experience in multiple MC roles	21 participants served in multiple MC roles over the years (e.g., regiment, garrison, Home Guard)
International deployments	11 participants had completed deployments to conflict zones
Length of service	From newly appointed MCs to up to 30 years
Average length of service	8.75 years (indicating a generally high level of professional experience within the sample)

To minimize the risk of identifying individual participants, demographic details such as gender and length of service are not reported in direct connection with quoted material. It should also be noted that the interview excerpts were selected to ensure a good gender balance between men and women, consistent with the overall distribution. The same precaution applies to other potentially identifying information, including specific deployments, ranks, duty stations, or particular events, which have been omitted or anonymized where necessary. Because all participants completed the survey at the same time and were aware of who participated, omitting such details was necessary to complicate back-tracing and reduce the risk of participants identifying one another. This consideration was particularly important given that the questionnaire was completed during a shared collective occasion. Although participants were offered the option to leave the room and respond in a different setting, the risk of mutual identification remained.

### Qualitative data coding procedure

The questionnaire consisted of 31 items organized into thematic sections and distributed across nine pages. When the allocated response space proved insufficient, participants were encouraged to continue writing on the reverse side of the questionnaire. In total, the dataset comprised approximately 450 pages of handwritten material, supplemented by a substantial number of additional pages. All material was subsequently transcribed into digital text files.

As an initial preparatory step, all handwritten questionnaire responses were transferred into Word documents. This digitization process was a prerequisite for enabling systematic analysis of the complete dataset using the qualitative data analysis software Atlas.ti.

The transcription process was organized by question rather than by respondent. Consequently, all responses relating to a specific question were compiled into a single document. To ensure analytical transparency and traceability, each response was labeled with a unique participant identifier. Although resource-intensive, this procedure was essential for maintaining a structured and consistent analytical workflow.

Once transcription was completed, the documents were imported into Atlas.ti for coding and further analysis. Because the questions contained clearly embedded concepts to which participants were expected to relate, such as formal tasks within the ACCES framework, an abductive analytical approach was employed.

An abductive approach involves moving iteratively between empirical observations and theoretical concepts in order to develop the most plausible explanation of the phenomenon under investigation, in this case how participants relate to a formal framework of tasks. Unlike deductive approaches, which begin with theory, and inductive approaches, which begin with data, abduction integrates both. The researcher starts from preliminary empirical observations, tests them against relevant theoretical perspectives, and then returns to the empirical material to refine the emerging interpretation. The theoretical component of the abductive analysis primarily consisted of the existing framework, that is, the task-governing structure. The participants’ empirical responses thus constituted the empirical material, which moved back and forth between an established theoretical structure and emerging insights into how participants experienced that structure. In this way, patterns and explanations develop through a continuous dialogue between data and theory (41–43).

Through abductive coding, an overall picture emerged of how the ACCES framework and its formally defined tasks are brought into ongoing dialogue with participants’ understandings of what they consider most important and meaningful for successfully carrying out their mission as chaplains.

The results of the analysis are presented according to this thematic internal structure, which unfolds the findings:

- The conversing MC
- The ceremonial representative
- Expertise in death and grief: Crisis support
- The educational MC: Category-based differences and discrepancies between time allocation and qualitative valuation
- Advising and supporting commanders and others
- Presence and time
- Key church-based competencies and resources

In the subsequent analysis, these seven themes serve as the principal organizing framework. The interpretation is continuously grounded in the empirical material through illustrative excerpts drawn directly from the coded questionnaire responses.

## Results

MCs devoted differing amounts of time to the various tasks within the ACCES framework. These differences were influenced by several factors. For example, depending on the category of military chaplain to which participants belonged, more or less time was available for the assignment. Variations were also related to how chaplains subjectively classified their work in ACCES terms, as well as to the needs of the units they served.

At regiments, air bases, naval stations, and garrisons where conscripts were trained over a full training year, the temporal scope of the assignment was different. It differed, for example, from that of a Home Guard battalion. In such units, service members serve only a few weekends per year, with the exception of any longer exercises. Training there places a strong emphasis on practicing profession-specific skills and the unit’s operational capability during short, intensive training periods. In addition, there were substantial variations in the amount of time allocated to chaplaincy work both within and between the different categories of military chaplains.

The **Tables 2** and **3** are based on mean values for the two overarching categories of military chaplains, derived from participants’ preliminary estimates of how their working time, expressed as percentages, was distributed across different tasks. These estimates provide an initial indication of how time is allocated within the ACCES framework, viewed against the background of the extensive experience of military chaplaincy that many participants possessed. The way in which the percentages are presented is also based on how the participants themselves chose to report the distribution of their time.

**Table 2:** Average distribution of time: regiments, air bases, naval stations, and garrisons MC.

Advisory role	Command support	Crisis support	Education	Conversation	Ceremonies	Other
8%	6%	11%	19%	31%	16%	9%

**Table 3:** Average distribution of time: Home Guard Battalion MC.

Advisory role	Command support	Crises support	Education	Conversation	Ceremonies	Other
14%	7%	7%	10%	35%	12%	15%

The estimated percentages should, however, be interpreted with caution, as they are based on subjective assessments. The illustration therefore needs to be understood in relation to the qualitative data, for example in terms of what participants experienced as most important and meaningful, as well as which tasks they had limited opportunities to carry out to any substantial extent.

### The conversing MC

Nearly all participants described conversations—soul care conversations—as one of the most important tasks in their role as military chaplains. Conversations can therefore be understood as a significant and deeply meaning-bearing pillar within the ACCES framework. This is also clearly reflected in the average percentage distributions shown above. Regardless of category, approximately one third of working time was devoted to the task of conversation.

The attributed importance of conversation should be understood as a broad umbrella term encompassing various conversational approaches that may have a soul care impact, create conditions for further dialogue and reflection, and cultivate trust and confidence. This may range from spontaneous conversations in barracks environments and in the field—addressing themes such as life, death, relationships, everyday concerns, faith, and ritual—to scheduled individual conversations with a more classical pastoral framing, clearly accompanied by the seal of confidentiality.

The following selection of participant voices illustrates how the task of conversation within ACCES was ascribed significance from different perspectives.

Participant 5 (Home Guard Battalion MC):

Conversations are experienced by me as the most important task.

Participant 15 (Home Guard Battalion MC), regarding conversational support both within and outside the military setting:

Support to individual service members is the most important and most common task. Several soldiers have contacted me with personal concerns. We have agreed on times and met for individual conversations in my home parish. Individual conversations are also the most common form during exercises.

Participant 16 (Home Guard battalion MC) on the need for conversation when life does not unfold as expected:

For the individual soldier, conversation is what matters most. Being able to offer support when life does not turn out as one had envisioned.

Participant 17 (Home Guard Battalion MC), on trust in conversations:

The conversations. That is where relationships and trust are built. That is what I draw on when some kind of crisis occurs.

Participant 21 (Home Guard Battalion MC), on different types of conversations:

The conversations! To both offload, support, and encourage. Sometimes to be a ‘trash can,’ and sometimes to reflect together on decisions, difficult situations, or simply to share joy and sorrow. The conversations are also about getting to know each other and getting to know God and God’s work in people’s lives.

Participant 36 (Regiments, Air Bases, Naval Stations, and Garrisons MC), on confidentiality and cultural competence:

The conversations. There is an opportunity to be open in conversations with me in a different way than when speaking to a psychologist or conscription counselor. Both because I have absolute confidentiality and because I hold a position in the organization where I am simultaneously part of it and yet somewhat outside it.

Participant 35 (Regiments, Air Bases, Naval Stations, and Garrisons MC), on conversational needs within the unit:

The conversations are important because it is clear that they are needed. I am approached when people [service members] want to book time.

Participant 37 (Regiments, Air Bases, Naval Stations, and Garrisons MC), on the moral significance of forward-positioned soul care:

Soul care and being far forward, where contact with soldiers and sailors becomes better, more natural, and more honest in their operational environment—often among those exposed to higher risks. Important for moral fighting power.

Participant 39 (Regiments, Air Bases, Naval Stations, and Garrisons MC), on conversations as a space for restoration:

Soul care—offering soldiers the opportunity to speak completely freely and unburden their hearts without the ‘risk’ of being judged or evaluated. Soul care becomes a breathing space for them, and their response is that they feel affirmed, seen, replenished, and able to leave the room with more hope, courage, and motivation than when they entered.

Participant 46 (position undisclosed), on the human need to articulate existential questions:

Conversations, soul care. Through conversation, I see the need for support in dealing with the fact that behind our uniforms we are also human beings—questions of life and death, faith, doubt, and relationships. That conversation, through small shifts in perspective, can make a difference. Confidentiality is extremely important.

The estimates of how much time Swedish military chaplains devoted to a conversational approach pointed in the same direction as the qualitative material. Within this material, conversations stood out as particularly prominent. Taken together, the Swedish military chaplain could be described as fundamentally conversational in character.

Skills and competencies for conversation across a wide range of forms and situations were distinctive. This included everything from everyday concerns to encounters with death and catastrophe. Conversations also served multiple functions. They could provide relief and function as a “pressure valve,” but also offer support, encouragement, courage, and hope, as well as foster relationships and trust. Confidentiality was of great importance for the conversations and constituted a key feature distinguishing MCs from mental health professionals within the armed forces, who may be subject to mandatory reporting requirements when an issue is assessed as affecting fitness for service. Conversations with a MC were therefore experienced as a safe haven, grounded in the assurance of absolute confidentiality.

### The ceremonial representative

The ceremonial dimension within the ACCES framework occupied a prominent position in the qualitative data, a finding that is also reflected in how MCs in both categories, on average, allocated their time to this area of practice. Ceremony constitutes a broad category encompassing, for example, the military worship service in the field known as Korum, various forms of memorial observances, and other traditional services such as Christmas Vespers and the Eucharist. In these contexts, the MC functions as a ceremonial representative and leader of a Church of Sweden tradition that has been embedded within military culture since the sixteenth century.

The following excerpts from a representative selection of participants illustrate how ceremonies were understood in terms of their significance and purposes within the ACCES framework.

Participant 3 (Home Guard battalion MC) on strengthening cohesion:

Training soldiers to participate in Korum and ceremonial formations aimed at strengthening cohesion and a shared value base.

Participant 5 (Home Guard battalion MC) on the task:

Ceremonial occasions are likewise perceived as important.

Participant 10 (Home Guard battalion MC) on providing existential resources through ritual:

Through ritual—the worship service—providing tools for engaging with and handling life.

Participant 11 (Home Guard battalion MC) on the function of Korum in the field:

Conducting Korum in the field to support and encourage the soldiers.

Participant 12 (Home Guard battalion MC) on the significance of ritual:

Ceremonies are rituals that are far more than words; they strengthen the sense of “we,” motivate, and provide a holistic understanding of the mission.

Participant 20 (Home Guard battalion MC) on the significance of symbols:

Ceremony is a way of building unit cohesion and providing symbols and language for experiences that can be difficult to articulate, such as during a memorial observance.

Participant 27 (Regiments, Air Bases, Naval Stations, and Garrisons MC) on the importance of establishing rites for resilience:

Rites and ceremonies play an important role in fostering resilience and recovery in war; they need to be established in times of peace.

Participant 33 (Regiments, Air Bases, Naval Stations, and Garrisons MC) on the importance of diverse church practices as part of ceremonial life:

Church practices such as the Eucharist, reflection groups, and Bible study groups.

Participant 35 (Regiments, Air Bases, Naval Stations, and Garrisons MC) on the significance of the Eucharist:

The Eucharist is important both as a way of building congregation and because it is evident that this moment is profoundly meaningful for those who participate.

Participant 37 (Regiments, Air Bases, Naval Stations, and Garrisons MC) on the depth of the ceremonial tradition:

Ceremony—Korum—hope, and a tradition that carries people regardless of belief or non-belief.

Participant 48 (position undisclosed) on the significance of ceremonies:

Ceremonies contribute to my visibility and to the building of unit cohesion.

Ceremonies of all kinds can, in contrast to conversations, be understood as having a distinctly collectivist and community-oriented character. Military personnel participated in these ceremonies in very large (battalion- or company-level) as well as smaller groups, engaging in a wide range of traditional rites, from prayers, religious services, and memorial observances to more explicitly group-oriented practices.

One of the central contributions of these ceremonies was their capacity to situate military personnel within a cultural framework and a tradition extending back several centuries—a tradition that articulated deeper meaning, purpose, and direction, while also offering existential interpretive resources for the circumstances in which they found themselves. In this way, the ceremonies sought to strengthen a sense of belonging to a broader cultural context, to one’s unit, and to provide care for the souls of military personnel from a wider pastoral perspective.

Ceremonies also served as occasions in which MCs became visible to many service members. This visibility had pedagogical significance, insofar as it enabled service members to gain knowledge of who the MC was, in cases where this had not previously been known.

### Expertise in death and grief: crisis support

Crisis support was regarded by many participants as an established and central task within the scope of their professional competence and expertise. Although crisis support did not account for a high proportion of their time allocation, this should be interpreted in light of the fact that, in peacetime, military personnel do not die or suffer serious injury on a daily basis. In other words, severe crises should not occur frequently when safety regulations are followed and military operations are well led—an expectation that is also reflected in the limited amount of time devoted to this task.

However, when crises do occur, the MC—whose primary professional identity is that of an ordained priest—becomes indispensable. It is therefore essential to interpret quantitative figures in relation to qualitative data.

What follows is a selection of participant statements illustrating the significance attributed to crisis support.

Participant 9 (Home Guard battalion MC), on death:

Going out to the units in the event of a death is something I see as very important, in order to provide groups with support and an opportunity to process and express their experiences.

Participant 22 (Home Guard battalion MC), on crisis support:

Questions and reflections concerning the meaning of life, purpose, and hope become especially pronounced in difficult situations.

Participant 26 (Regiments, Air Bases, Naval Stations, and Garrisons MC), on responses to crisis support:

Crisis support seems to be the area where I perceive the strongest response—positive responses.

Participant 29 (Regiments, Air Bases, Naval Stations, and Garrisons MC), viewing this as a competence uniquely shaped by church tradition:

Crisis support, including memorial services. This is the church’s specific contribution, grounded in my competence, identity, and experience as a priest.

Participant 34 (Regiments, Air Bases, Naval Stations, and Garrisons MC), on need:

Crisis support—when crises and accidents occur, there is a great need.

Participant 37 (Regiments, Air Bases, Naval Stations, and Garrisons MC), on crisis support in life and death:

Crisis support! We take care of one another, leave no one behind, and stand together. Dignity in both life and death.

Participant 46 (position undisclosed), on competence and demand:

Crisis support—I notice that my professional experience of dealing with and supporting people in matters of death and crisis is in demand and clearly needed.

There are various forms of crisis support. In this context, however, the primary focus was on death. Within a Swedish context, Church of Sweden has long held a particular responsibility for matters relating to death, grief, and burial. Historically, priests have been educated and trained to accompany individuals in the most vulnerable situations of life, not least in the event of death. To this day, the Church of Sweden serves as the principal authority for funeral services in most parts of the country.

This longstanding responsibility has resulted in a deep and consolidated body of knowledge concerning dying, processes of grief, and funerary rituals. Such expertise constitutes a distinctive and unique competence among Swedish military chaplains who are also ordained priests. They combine an understanding of the military context with professional experience in addressing death and grief—an integration of skills that is of particular importance in crisis and disaster situations.

### The educational MC: category-based differences and discrepancies between time allocation and qualitative valuation

Within the ACCES framework, educational initiatives also form an important component of MC practice. These include education in areas such as morality, ethics, values-based exercises, rules of conduct for soldiers, the law of armed conflict, death, and war graves services, among others. Education was likewise described as a significant task in participants’ estimations of how they allocate their time. There is clearly an educational dimension present among both major categories of MCs; however, notable differences emerge in how this task is perceived and valued.

According to estimations of time distribution, MCs serving at regiments, air bases, naval stations, and garrisons reported spending considerably more time on educational activities, and doing so more frequently, than MCs serving within Home Guard battalions. Within the group of MCs serving at regiments, air bases, naval stations, and garrisons, reported time allocation aligned closely with the qualitative valuation of education as an important and meaningful task.

By contrast, the qualitative data showed that Home Guard battalion MCs rarely referred to education when asked about their most important tasks, with the exception of a single participant who mentioned rules of conduct for soldiers. At the same time, several Home Guard battalion MCs expressed a desire to engage more extensively in educational work. Taken together, these findings indicate a discrepancy within the Home Guard battalion group between reported time allocation and the qualitative valuation of education in terms of what is perceived as most important and meaningful.

The following excerpts, primarily drawn from MCs serving at regiments, air bases, naval stations, and garrisons, illustrate how education was understood as a core task.

Participant 27 (Regiments, Air Bases, Naval Stations, and Garrisons MC) on the dimensions of education:

Providing education challenges me to deepen my own knowledge in a given area. This also creates visibility toward soldiers and conscripts.

Participant 31 (Regiments, Air Bases, Naval Stations, and Garrisons MC) on education as meaningful work:

I believe I am most useful when I teach, as I provide soldiers with tools to care for themselves and for one another.

Participant 34 (Regiments, Air Bases, Naval Stations, and Garrisons MC) on the deepening dimension of education:

Education—making visible the effects of weapons on body and soul.

Participant 34 (Regiments, Air Bases, Naval Stations, and Garrisons MC) on the preparatory character of education:

Education concerning death, ethics, and morality in order to be prepared for heightened readiness and/or war.

Participant 46 (position undisclosed) on the universal dimension of education:

Education is important because the subjects I teach concern everyone, regardless of belief, on a human level.

As noted earlier, differences in how education was valued may partly be explained by structural conditions. MCs serving at regiments, air bases, naval stations, and garrisons often have more time available for their assignment, and these units are significantly larger than Home Guard battalions. Moreover, they are, by their nature, engaged throughout the annual cycle in training activities, such as the education of conscripts. In addition, MCs in these settings tend to have greater autonomy in shaping their role compared to MCs serving in Home Guard battalions.

By contrast, Home Guard battalions constitute standing units that can be deployed on short notice and that conduct joint training during a limited number of days each year. This results in a high operational tempo with a strong emphasis on skills training and functional readiness among soldiers, commanders, and units alike. MCs within Home Guard battalions may also be assigned to staff roles, which further binds them to other duties. Constraints related to time, as well as limited awareness among commanders, may thus contribute to differences in how MCs are utilized for educational purposes. These factors may also influence how MCs are employed in other ACCES-related tasks.

At the same time, crisis support illustrates the interactive nature of the ACCES framework, as it also encompasses various forms of ceremonies, such as funerals and memorial services.

### To advise and support commanders and others

In theory, the two tasks commonly referred to as the advisory role and command support may appear easy to distinguish, but in practice they are not always as clearly separable. Whether participants, when estimating their time allocation, were able to discern what belonged to advising and what constituted support remains an open question. With regard to command support, both categories of MCs estimated their time investment in very similar ways, and for several participants this form of support was closely associated with crisis support, that is, situations involving death.

By contrast, time devoted to the advisory role was estimated to be higher within a Home Guard battalion context. This may be related to the character of this type of unit, which typically consists of older, working professionals serving part-time. In such units, everyday life in the field is often shared more closely, which may contribute to MCs being approached more frequently for advice.

There is no doubt that commanders seek both support and advice from MCs. However, the qualitative data reveal several nuances in participants’ responses that do not always make it entirely clear whether a given activity should be understood as support or advice. Nor are the definitions of these tasks exhaustive or definitive. Ultimately, it was left to the participants themselves to decide under which category they estimated the time they had spent.

For example, Participant 6 (Home Guard battalion MC) described command support and crisis support as an important and meaningful task:

Command support and crisis support—being the commander’s ear to the ground. Listening in, conveying perspectives from the soldiers. Helping the commander make wise decisions. Helping the unit function better in relation to its mission.

Participant 16 (Home Guard battalion MC) commented on the overlap between the tasks:

Advising and command support overlap and are difficult to distinguish, but they may be significant for the unit as a whole.

Participant 20 (Home Guard battalion MC) described command support as follows:

Support to commanders in personnel matters, in order to give them a broader picture of events and needs, and to help those I support function well in their roles.

Participant 37 (Regiments, Air Bases, Naval Stations, and Garrisons MC) reflected on the indirect and direct significance of advising:

Advising, indirectly. It is rare that it is explicitly requested, but in conversations my perspective and knowledge are taken into account, and certain activities are changed as a result of my advice.

Advising and support can be understood as distinct tasks, but they may also overlap and be difficult to discriminate between. It can be assumed that an MC is regularly called upon to support commanders and units in situations involving, for example, deaths, where the boundaries between tasks are more easily identified. The advisory role, by contrast, is often more diffuse, operating on both formal and informal levels and encompassing a broad existential spectrum, from questions of life, spirituality, the church, and religion to ethics, morality, and wider understandings of the human condition. The extent to which advice is sought is plausibly related to factors such as the perceived competence of the MC, the level of trust placed in them, the type of unit in which the MC serves, the organizational level at which the MC operates (for example, whether the MC serves as a regional MC or works part-time at a regiment), the relationship between commanders, service members, and the MC, and whether the MC shares field life with the soldiers, including sleeping and living alongside them during exercises.

When the percentages allocated to advising and support are combined, a relatively substantial portion of time is devoted to these tasks (14% for MCs at regiments, air bases, naval stations, and garrisons, and 21% for Home Guard battalion MCs). Nevertheless, in the qualitative responses these tasks are not accorded the same degree of importance or perceived meaningfulness as conversations, ceremonies, education, and crisis support.

### Presence and time

Many participants emphasized that one of the most important aspects of their work—an aspect not explicitly included among the formal tasks of the ACCES framework—was the relational dimension, particularly expressed through presence. A small number of participants stated quite plainly that relationship-building constituted the most central task, as the other responsibilities could not be carried out to any significant extent without it.

Three representative voices illustrate this emphasis:

Participant 11 (Home Guard battalion MC):

Establishing contacts with everyone in the battalion, so that one does not become ‘the man in a box’ who only appears when accidents, deaths, or debriefing conversations are needed.

Participant 32 (Regiments, Air Bases, Naval Stations, and Garrisons MC):

Building relationships, walking alongside conscripts, officers, and personnel, sharing their everyday lives. On that foundation, I can then provide support in a credible and natural way in pastoral conversations, advising, command support, and crisis support.

Participant 38 (Regiments, Air Bases, Naval Stations, and Garrisons MC):

Presence, relationship-building! Being there when things happen. Being the extended hand of the church. If we have built strong relationships during times when ‘all is well,’ it becomes much easier to be of help when a crisis arises.

More than half of the participants reported working beyond the percentage of time formally allocated to their assignment across all categories, but this was particularly pronounced among Home Guard battalion MCs. This group typically had approximately 8–10 contracted days per year, which they experienced as insufficient to maintain a level of presence they considered satisfactory. This limitation was closely related to the perceived need to build relationships through sustained presence in daily activities and during exercises, as well as to respond to needs and requests from units, commanders, and individual service members alike.

Through presence, relationships, trust, legitimacy, and cultural understanding of the unit are established, while the MC simultaneously becomes familiar with both soldiers and commanders.

### The most important church-based competencies and resources

With regard to which church-based and knowledge-related resources and tools MCs drew upon, these were clearly reflected in how participants assessed time allocation, perceived importance, and experienced meaningfulness in relation to the tasks outlined in the ACCES framework.

The entire theological education culminating in ordination, together with the accumulated experience of serving as parish priests and meeting people in conversation across all life situations, emerged as an immensely important asset. This was closely connected to the task of conversations and to the confidentiality inherent in the pastoral role, which functioned as a clear demarcation between MCs and mental health care professionals. It should also be noted that the Church of Sweden is an open national church, with more than half of the population as members. As a result, priests generally encounter a broad cross-section of society and engage in a form of general and existential soul care.

Participant 22 (Home Guard battalion MC) articulated this shared emphasis on competence and experience:

Competence and support derived from having conducted many pastoral care conversations and grief conversations as a parish priest. Daring to meet the person where they are. Daring to hear, listen to, and encounter pain, and still being able to offer hope—or, for a moment, to serve as a substitute for hope.

The second emphasized area of knowledge and experience stemmed from the extensive exposure to death, grief, and funerals that all priests had encountered to a significant degree. This corresponded closely to the task of crisis support.

The following voices embody this church-rooted competence:

Participant 8 (Home Guard battalion MC):

My parish experience of encountering and working in situations of grief and crisis.

Participant 28 (Regiments, Air Bases, Naval Stations, and Garrisons MC):

My familiarity and experience with death, with the bereaved, and with moving toward hope.

Participant 37 (Regiments, Air Bases, Naval Stations, and Garrisons MC):

Crisis support—death—linked to the fact that, as priests, we are trained in crisis management and funerals.

The third resource concerned ecclesial rituals, encompassing deeply rooted traditional practices, worship services, the sacraments, prayer, and blessing. This corresponded to the task of ceremonies.

The following participants gave voice to a perspective that echoed across the wider group of participants, highlighting the importance of this church-provided resource:

Participant 12 (Home Guard battalion MC):

Knowledge of the significance of ritual in creating coherence and meaning.

Participant 17 (Home Guard battalion MC):

Knowledge and experience of the significance and power of ritual.

Participant 29 (Regiments, Air Bases, Naval Stations, and Garrisons MC):

Prayer, hymns, the liturgical order and treasury of worship, biblical texts, and a language for the extreme edges and boundary situations of existence.

Participant 40 (Regiments, Air Bases, Naval Stations, and Garrisons MC):

Competence and habitual practice in using ritual.

Together, these constituted the three major thematic areas of church-based competence and resources, corresponding to the tasks of conversations, crisis support, and ceremonies.

## Discussion

Several important takeaways emerge from this study.

### Time allocation

Not least the two empirical mean-value illustrations depicting how time is perceived to be distributed across ACCES-related tasks within the two major categories of MCs.To the best of our knowledge, no such illustrations have previously existed in a research context. These figures should be understood as participants’ experiential estimations—ideas grounded in lived experience—of how time is allocated among tasks. Such distributions are, of course, subject to change over time. For example, in the event of war, crisis support would reasonably be expected to increase dramatically due to large numbers of service members being killed or severely injured, a development also documented in research from wartime Ukraine (44). In peacetime, by contrast, the need for crisis support is relatively limited. How these time distributions would compare internationally is difficult to determine, as comparable studies are lacking. Moreover, the frameworks and institutional regulations governing MCs’ employment conditions and task assignments vary across countries and are not easily comparable (16, 21–23).

The second takeaway concerns ACCES, the Swedish framework for Military Soul Care, which specifies the tasks MCs are expected to perform. Normative frameworks of this kind regulate—more or less strictly—MCs’ functions, responsibilities, and limitations within military organizations (1, 16, 21–23).

### Building trust beyond ACCES: The centrality of presence

In the Swedish context, the ACCES task structure largely reflects what MCs report doing in practice, both in terms of concrete activities and perceived meaningfulness. However, the qualitative data identified one activity not explicitly included in the framework: the importance of presence within the organization for the purpose of building relationships and establishing trust. Previous research has consistently shown that military chaplaincy is characterized by presence—often described as a *ministry of presence*—within everyday military life, and by work conducted in close proximity to units’ training and operational activities (1, 12–15). Without a presence that fosters trust and relationships, it is difficult to carry out chaplaincy tasks—to chaplain service members—effectively. This was a key reason why many participants reported working well beyond their formal contractual hours, particularly among Home Guard battalion MCs, but also across other categories.

This can be viewed from several perspectives. Competencies alone are not sufficient in a military context, as military chaplaincy also requires a certain degree of presence in terms of time in order to build relationships and trust (13–15). There are no shortcuts. Presence is best understood as a prerequisite for carrying out the professional duties articulated as ACCES. Consequently, MCs are willing to devote unpaid personal time to achieving the level of *ministry of presence* they consider necessary to carry out their tasks effectively. This, in turn, is partly a contractual and employer-related issue, where both the Church of Sweden and the Swedish Armed Forces could jointly identify ways forward. For example, the Armed Forces could allocate additional funding to the Church of Sweden, enabling increased service time for certain categories of MCs. Some contracts—for instance those governing Home Guard battalion MCs—could also be revised to include more contracted days. This could be more readily justified by introducing an additional formal task explicitly related to the need for demonstrated presence (through participation) within military units.

However, rather than constituting an additional professional duty, presence should be understood as the social and relational precondition necessary for the effective enactment of the formal duties articulated within ACCES. In this sense, presence functions as a form of social and relational lubricant that enables these duties to be carried out effectively. When such tacit knowledge is not formalized as a distinct task, there is a risk that the considerable time it requires remains unrecognized by employers who primarily attend to the time needed to complete formalized duties. This challenge may be addressed either by fostering greater organizational awareness of the time required to sustain presence or by formally recognizing presence itself as a professional duty.

### Task saliency and internal hierarchies within ACCES

The third takeaway concerns the apparent existence of an internal hierarchy of needs and perceived meaningfulness in relation to the tasks outlined in ACCES. This hierarchy is reflected in the mean-value models (e.g., conversations) but not consistently so (e.g., crisis support).

Conversations emerge as the most important task and should be understood as a very broad and inclusive practice, ranging from informal conversations over coffee in barracks or in the field to scheduled individual soul care conversations conducted under the seal of absolute confidentiality. MCs operate in a context characterized by particular stressors, such as the use of violence, ethical dilemmas, loss, separation, and existential vulnerability (4–6). Conversations can be understood as a preventive and stabilizing form of support that contributes to personnel’s long-term psychological, social, and existential sustainability, addressing spiritual, ethical, moral, human, ecclesial, religious, and spiritual concerns (5, 7, 8, 10, 11). In addition, conversations functioned as a method or strategy for establishing and strengthening relationships and trust among military personnel. Conversational skills constitute a core professional competence among Swedish MCs, developed through formal training and further refined in the context of parish ministry. However, for these skills to be applicable and effective within a military setting, they must be complemented by military cultural competence and a contextual understanding of military life and organizational culture. It should also be noted that conversation, broadly understood—from sitting more or less silently during informal interactions (e.g., over coffee) to more structured conversations—may in some respects conceptually overlap with the notion of being present, even though participants tended to describe this in terms of conversing.

Ceremonies were also experienced as highly important and meaningful, as was crisis support. These three tasks can be said to reflect priestly identity and the exercise of priestly ministry in a Swedish context particularly well, where soul care conversations, various forms of worship, ecclesiastical rites and rituals, as well as death, grief, and funerals constitute part of a priest’s everyday work within the Church of Sweden (27). A defining characteristic of these competencies is that they are rooted almost entirely in extensive experiential learning derived from the everyday practices of parish ministry. In the Swedish context, the expectation of long-term parish service constitutes a key prerequisite for appointment as a MC. At the same time, such competencies require ongoing cultivation and maintenance in order to sustain the quality, credibility, and integrity of the chaplaincy role (27). In this respect, the shared chaplaincy appointments common in Sweden—where chaplains combine military service with continued parish work—may be seen as a particular strength.

Teaching is likewise an important dimension of the priestly vocation in the Church of Sweden and was also considered an important task in a military context. However, this was relatively dependent on the type of MC role. Several Home Guard battalion MCs reported that time constraints, prioritization of skills training and exercises, and limited awareness of how MCs could be utilized reduced their opportunities to engage in educational activities. Clearer information and increased knowledge regarding how commanders within the Swedish Armed Forces can make use of MCs in educational contexts—particularly in relation to ethics, morality, and values-based issues (1, 5, 18, 19)—may constitute an effective measure for enhancing the impact of MCs in the Swedish setting. In particular, ongoing academic professional development initiatives aimed at equipping Swedish MCs with a deeper understanding of identity and moral injury in a military context appear especially important (45). It is therefore essential that as many MCs as possible participate in, and complete, such educational initiatives.

Participants also devoted a considerable amount of time to command support and advisory roles, although the qualitative data suggest that these were not experienced as the most meaningful tasks. Research, however, on MCs in Ukraine indicates that this role may become more significant in wartime, when MCs also function as defenders of human values and humanity within the context of war (44).

### Professional flexibility in military contexts: identity, culture, and competence

The fourth takeaway concerns the clear movement between different roles: the role of general conversation partner, the role of conversation partner under conditions of absolute confidentiality, the role of ceremonial leader, the role of crisis support specialist, the role of educator, the role of advisor, and the role of staff member or similar. From a professional perspective, this places substantial demands on flexibility. An MC must possess a strong capacity to move across multiple expert positions and activate different types of competencies in highly diverse situations. This also includes sufficiently mastering a military identity, military cultural understanding, and contextual competence in order to be perceived as relevant. This is closely connected to the previously discussed temporal dimension and the importance of presence.

The ecclesial priestly identity, tools, and competencies were decisive for the MC mission, with particular emphasis on expertise related to death, grief, and funerals when crises arose. This competence becomes even more salient in the event of war, where the reality of death is both unavoidable and extensive. Research from Ukraine indicates that this was experienced as the most emotionally demanding area of work for Ukrainian MCs (44). This is something both MCs and military organizations must prepare for and train in during peacetime in order to be adequately equipped in the event of war.

## Concluding remarks

In sum, this study demonstrates that while the ACCES framework largely reflects the formal structure of military chaplaincy tasks in Sweden, it does not fully capture the relational and temporal conditions required for their effective enactment. Most notably, presence emerges as a foundational yet under-recognized dimension that enables the performance of all other tasks.

**Figure 1:**
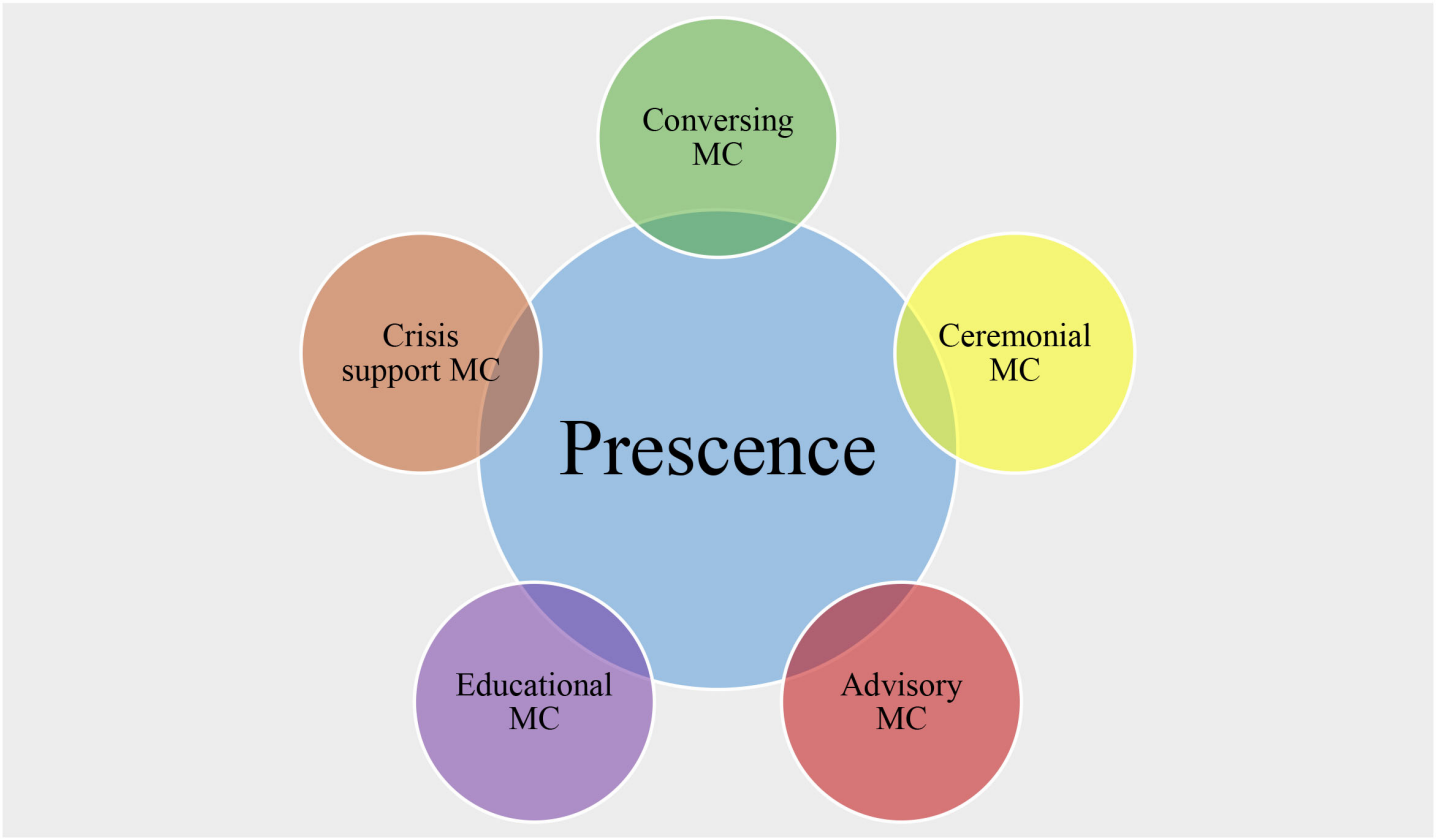
Presence and ACCES: A conceptual model of military chaplaincy.

The findings also point to an internal hierarchy of perceived meaningfulness and a high degree of role flexibility, placing significant demands on chaplains’ professional competence and time, as illustrated in **Figure 1**, with the more meaningful tasks positioned higher in the figure.

Together, these results highlight the need to more explicitly acknowledge the relational and temporal dimensions of chaplaincy work, both within organizational frameworks and in future research.

### Limitations and future research

One limitation of the qualitative method employed in this study is that it did not allow for follow-up questions. Once responses had been submitted, the researcher was unable to return to individual participants should additional questions arise. Future research should employ in-depth interviewing techniques to facilitate greater interaction (46).

Another limitation is that participants were sometimes brief in their responses; however, it should be noted that interviewees may also express their views succinctly while still conveying their intended meaning. Again, the use of in-depth interviewing techniques involving additional interaction may have helped to build greater rapport and elicit richer data.

Regarding the mean values used in the illustrations of how time is distributed across different tasks, it should be emphasized that these are based on participants’ subjective estimations. In other words, no logged or objective time-use data were available. It should also be noted that for other types of MCs in a Swedish context—for example at the regional level or during deployment—the distribution of time in relation to tasks may differ. In such contexts, additional tasks may also emerge, but these fall outside the scope of the present study. Furthermore, as the figures represent mean values, the distribution of time may be experienced quite differently at the individual level within each category. As previously noted, the allocation of time across tasks may also change radically if the situation shifts, for example in the context of an operation or a war scenario.

Future research could, for instance, employ a longitudinal mixed-methods design in which participants from different MC categories are equipped with structured logbooks over the course of a year in order to gain a more precise and ecologically valid understanding of how time is distributed across tasks. The logbooks could include predefined task categories aligned with ACCES, alongside open fields for emergent activities, allowing for both quantitative aggregation and qualitative nuance. This design could be complemented by semi-structured interviews conducted at multiple time points, enabling a more in-depth exploration of how the urgency, perceived meaningfulness, and situational need of tasks relate to MCs’ lived everyday practice. Such a design would allow for triangulation between recorded time use, narrative accounts, and contextual changes, thereby strengthening both the validity and interpretive depth of future research on military chaplaincy.

## Data Availability

The original contributions presented in the study are included in the article/supplementary material, further inquiries can be directed to the corresponding author/s.
